# *SNAI2/Slug* gene is silenced in prostate cancer and regulates neuroendocrine differentiation, metastasis-suppressor and pluripotency gene expression

**DOI:** 10.18632/oncotarget.2736

**Published:** 2015-02-18

**Authors:** Silvia Esposito, Marco V. Russo, Irma Airoldi, Maria Grazia Tupone, Carlo Sorrentino, Giulia Barbarito, Serena Di Meo, Emma Di Carlo

**Affiliations:** ^1^ Department of Medicine and Sciences of Aging, Section of Anatomic Pathology and Molecular Medicine, “G. d'Annunzio” University, Chieti, Italy; ^2^ Ce.S.I. Aging Research Center, “G. d'Annunzio” University Foundation, Chieti, Italy; ^3^ Specialisation School in Clinical Biochemistry, “G. d'Annunzio” University, Chieti, Italy; ^4^ Laboratory of Oncology, Istituto Giannina Gaslini, Genova, Italy

**Keywords:** Prostate Cancer, Neuroendocrine Differentiation, Laser Capture Microdissection, SNAI2/Slug

## Abstract

Prostate Cancer (PCa)-related deaths are mostly due to metastasization of poorly differentiated adenocarcinomas often endowed with neuroendocrine differentiation (NED) areas.

The *SNAI2/Slug* gene is a major regulator of cell migration and tumor metastasization. We here assessed its biological significance in NED, and metastatic potential of PCa.

SNAI2 expression was down-regulated in most PCa epithelia, in association with gene promoter methylation, except for cell clusters forming: a. the expansion/invasion front of high-grade PCa, b. NED areas, or c. lymph node metastasis.

Knockdown of *SNAI2* in PC3 cells down-regulated the expression of neural-tissue-associated adhesion molecules, Neural-Cadherin, Neural-Cadherin-2, Neuronal-Cell-Adhesion-Molecule, and of the NED marker Neuron-Specific Enolase, whereas it abolished Chromogranin-A expression. The metastasis-suppressor genes, Nm23-H1 and KISS1, were up-regulated, while the pluripotency genes SOX2, NOTCH1, CD44v6, WWTR1/TAZ and YAP1 were dramatically down-regulated. Over-expression of *SNAI2* in DU145 cells substantiated its ability to regulate metastasis-suppressor, NED and pluripotency genes. In PCa and lymph node metastasis, expression of SOX2 and NOTCH1 was highly related to that of SNAI2.

In conclusion, I. *SNAI2* silencing in PCa may turn-off the expression of NED markers and pluripotency genes, while turning-on that of specific metastasis-suppressors, II. SNAI2 expression in selected PCa cells, by regulating their self-renewal, NED and metastatic potential, endows them with highly malignant properties. SNAI2 may thus constitute a key target for modern approaches to PCa progression.

## INTRODUCTION

The metastatic spread is responsible for most PCa-related deaths. A key event during its early phases is the reactivation of a latent embryonic program, known as epithelial-to-mesenchymal transition (EMT), whereby transformed epithelial cells acquire mesenchymal traits and migratory ability. *SNAI2*, also known as *Slug* or *SNAIL2* gene, plays a major role in this complex cell and genetic program [1, 2].

*SNAI2* encodes a zinc-finger protein of the Snail family of transcription factors. During embryonic development, SNAI2 is expressed in the dorsal neural tube [3] and drives EMT which leads to mesodermal and neural crest cell migration [4]. In post-natal life, the *SNAI2* gene is widely expressed in adult human tissues, including the prostate [5, 6], while its amplification [1] or interaction with specific oncogenes [5, 7] have been demonstrated in a wide spectrum of human cancers.

Studies performed in cell and human tumor xenograft models of PCa have revealed that SNAI2 promotes cell migration and invasion and is a critical mediator of Cyclin D1b–induced oncogenic activity [8, 9]. It has been recently found that Slug is critically involved in determining the stem cell state of mammary cancer [10]. This property, in turn, may condition the differentiation of cancer and correlate with its aggressiveness [11, 12].

Since the histological hallmark of aggressive PCa is a poorly differentiated glandular architecture of high Gleason grade and presence of nests of neuroendocrine cells, namely neuroendocrine differentiaton (NED) areas [13, 14], it may be supposed that SNAI2's role in prostate carcinogenesis involves the regulation of stem cell- or NED-associated genes.

We here investigated the *SNAI2* expression profile in microdissected PCa foci with different grades of differentiation, established the mechanisms underlying its regulation, and revealed new aspects of its implications in the differentiation and malignant evolution of PCa.

## RESULTS

### *SNAI2* gene expression level was down-regulated in neoplastic epithelia of both well and poorly differentiated PCa

**Figure 1:**
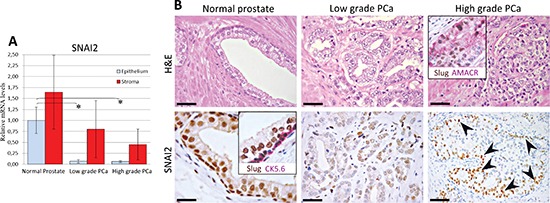
Expression of SNAI2 in normal and neoplastic prostate tissue **(A) *SNAI2* mRNA expression in normal and cancerous prostate tissues from PCa patients.** Histogram representing the relative expression ± SD of *SNAI2* mRNA in microdissected histologically normal epithelium and stroma, and their neoplastic counterparts with low or high Gleason grades, from prostatectomized patients (groups of 15), normalized with the housekeeping gene HPRT. One-way ANOVA for comparisons between epithelial compartments of normal prostate, low- and high-grade PCa: *p* < 0.0001. *p < 0.01 Tukey HSD Test compared with normal prostate epithelium. **(B) SNAI2 protein expression in normal and cancerous prostate tissues from PCa patients.** Prostate tissue from PCa patients displayed a bright SNAI2 nuclear expression in both luminal and basal epithelial cell layer as confirmed by the double SNAI2(brown)/CK5.6(fuchsia) staining (inset in the bottom left panel). SNAI2 expression was frequently lost in malignant epithelium of PCa with either low or high Gleason grades. A strong SNAI2 expression was confined to neoplastic cells bordering high-grade PCa foci, as revealed by double SNAI2(brown)/AMACR(fuchsia) staining (inset in the upper right panel) and indicated by arrowheads in the single immunostained section (bottom right panel). Magnification: X630, bottom left panel; X200, bottom right panel; X400, the remaining panels. Scale bars: bottom left panel, 20 μm; bottom right panel, 50μm, the remaining panels, 30 μm.

To clarify the biological functions of SNAI2 in human prostate carcinogenesis, we first assessed its expression levels in cell populations microdissected from both epithelial and stromal compartments of PCa foci with low (well differentiated) or high (poorly differentiated) Gleason grade (≤ 3 versus > 3), and from their histologically normal counterparts harvested far from the cancer (Figure 1A and 1B). The mean level of *SNAI2* mRNA of malignant stroma was not significantly different from that of the normal counterpart, whereas it was considerably (*p* < 0.05) down-regulated in the neoplastic epithelial cell populations from both low- (~14 times) and high- (~16 times) grade PCa (with no appreciable difference between them) (Figure 1A), in comparison with the normal epithelium.

*SNAI2* expression levels in the normal prostatic epithelium and stroma were comparable to those found in normal epithelium and stroma from control patient samples. No significant association was disclosed by the Mann-Whitney U or the χ^2^ test between SNAI2 expression levels and any of the clinical and pathological parameters (Supplemental Tables S1–S3).

**Figure 2:**
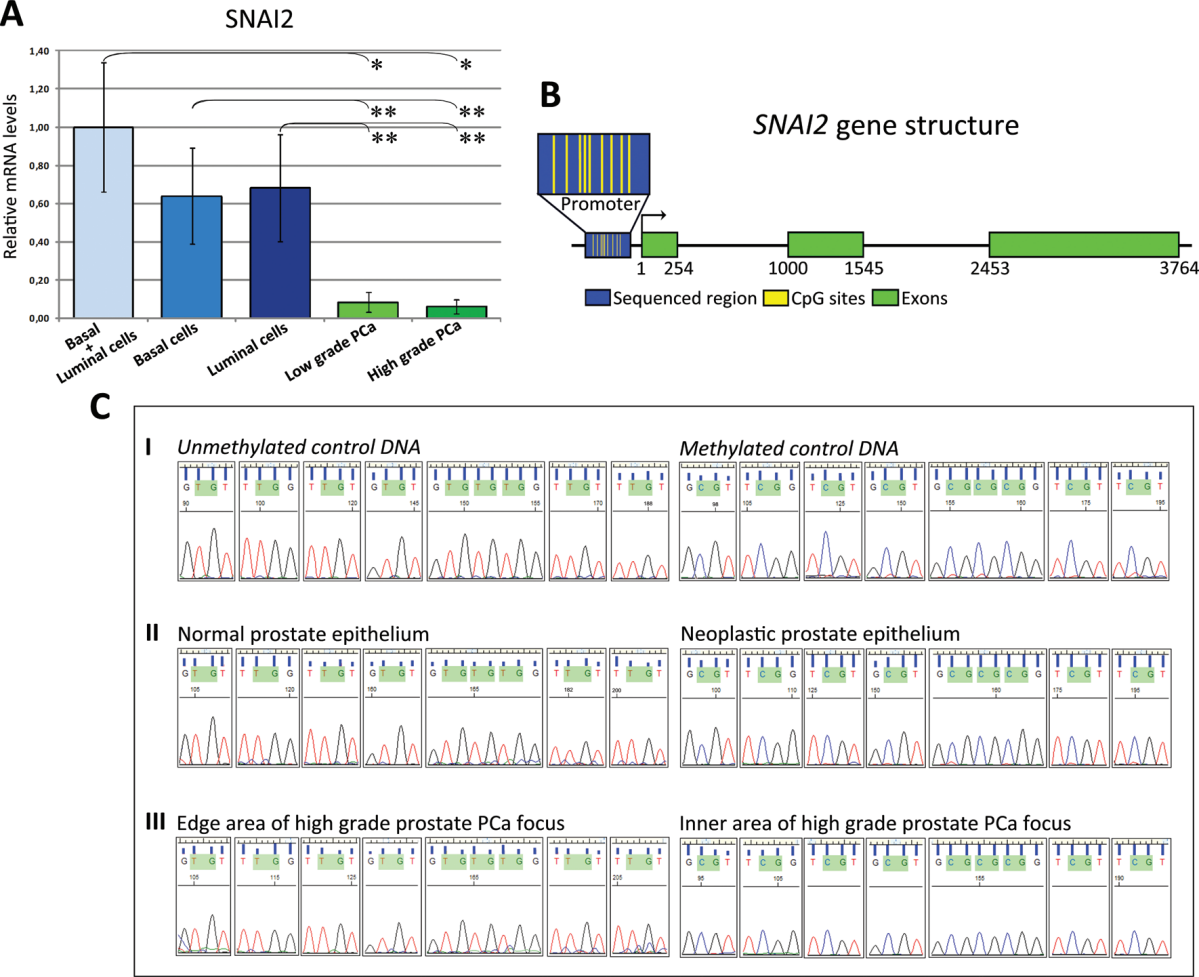
Expression of *SNAI2* in normal prostate epithelium and bisulfite genomic sequencing of the proximal promoter region of the SNAI2 gene **(A) *SNAI2* mRNA expression in the basal and luminal epithelial cells of normal prostate glands.** Histogram representing the relative expression ± SD of *SNAI2* mRNA in microdissected histologically normal basal and luminal secretory epithelium (15 randomly chosen normal samples from PCa patients) and neoplastic epithelium from low and high-grade PCa. One-way ANOVA for comparisons between normal basal and luminal secretory epithelium and neoplastic epithelium from low and high-grade PCa: *p* < 0.0001. *p < 0.01 Tukey HSD Test compared with low or high-grade PCa. **p < 0.05 Tukey HSD Test compared with low or high-grade PCa. **(B) DNA methylation status of *SNAI2* gene promoter in PCa.** The *SNA12* gene promoter (blue box) contains 9 sparsely spaced CpG islands (small yellow bars) in its central portion. **(C) Genomic sequencing of the proximal promoter region located 130 bases upstream from the TSS of the *SNAI2* gene in microdissected normal and cancerous prostate epithelium, or microdissected cells from the inner and edge areas of high-grade PCa foci.** I. The electropherogram shows the methylation status of each CpG island in control DNA where **TG** represents the unmethylated site (top left), and **CG** represents the methylated site (top right). II. CpGs were unmethylated in normal prostate epithelium (middle left) and methylated in neoplastic prostate epithelium (middle right). III. CpGs were unmethylated in cell clusters from the edge area (bottom left), and methylated in cell clusters from the inner area of the high-grade PCa focus (bottom right).

To determine whether the lack of *SNAI2* expression in the epithelial compartment of PCa was due to absence of the basal cell layer [15], we analyzed *SNAI2* expression in distinctly microdissected basal and luminal epithelial components. Since *SNAI2* expression levels were not significally different within basal cells, luminal secretory cells and the overall glandular epithelia (Figure 2A), it was evident that *SNAI2* down-regulation in PCa was unrelated to this basal cell loss. Similar data were obtained from normal samples of control patients.

### Malignant epithelia from PCa mostly displayed *SNAI2* gene promoter methylation

Since DNA methylation marks the entire spectrum of prostate carcinogenesis [16], we next tested its involvement in the loss of *SNAI2* gene expression in PCa. Because DNA methylation surrounding the transcription start site (TSS) is closely associated with transcription activity, we performed sodium bisulfite genomic sequencing of the proximal promoter relative to a section of 309 bp located 130 bases upstream from the TSS. This promoter region contains 9 sparsely spaced CpGs in its central portion (Figure 2B). They were unmethylated in normal prostate epithelial cells (from normal samples of both PCa and of control patients), and methylated in the majority of neoplastic cell foci with low- (74%) and high- (80%) grade feature (Figure 2C). These data strongly suggest that the SNAI2 down-regulation observed in most PCa epithelia is linked to hypermethylation of the SNAI2 gene promoter.

### SNAI2 expression characterized cancer cell clusters at the invasion/expansion front and NED areas of high-grade PCa, and lymph node metastasis

SNAI2 expression was immunohistochemically assessed to validate molecular data at the protein level, and also visualize any fluctuation (suggested by the lack of methylation in some of the microdissected cancer cell populations) of its expression within the heterogeneous prostate tissue.

In the stromal compartment of both normal and cancerous prostate tissue, SNAI2 expression was detectable in small mononuclear-like leukocytes, and cells endowed with fibroblast morphology.

The normal glandular epithelium displayed a bright nuclear expression of SNAI2 in both the luminal secretory cells Cytokeratin (CK)18+ and the basal cell CK5.6+ layer (Figure 1B), whereas the neoplastic epithelium (alpha-methylacyl-CoA racemase, AMACR+), from both low- and high-grade PCa foci, was mainly devoid of SNAI2 expression (Figure 1B).

**Figure 3:**
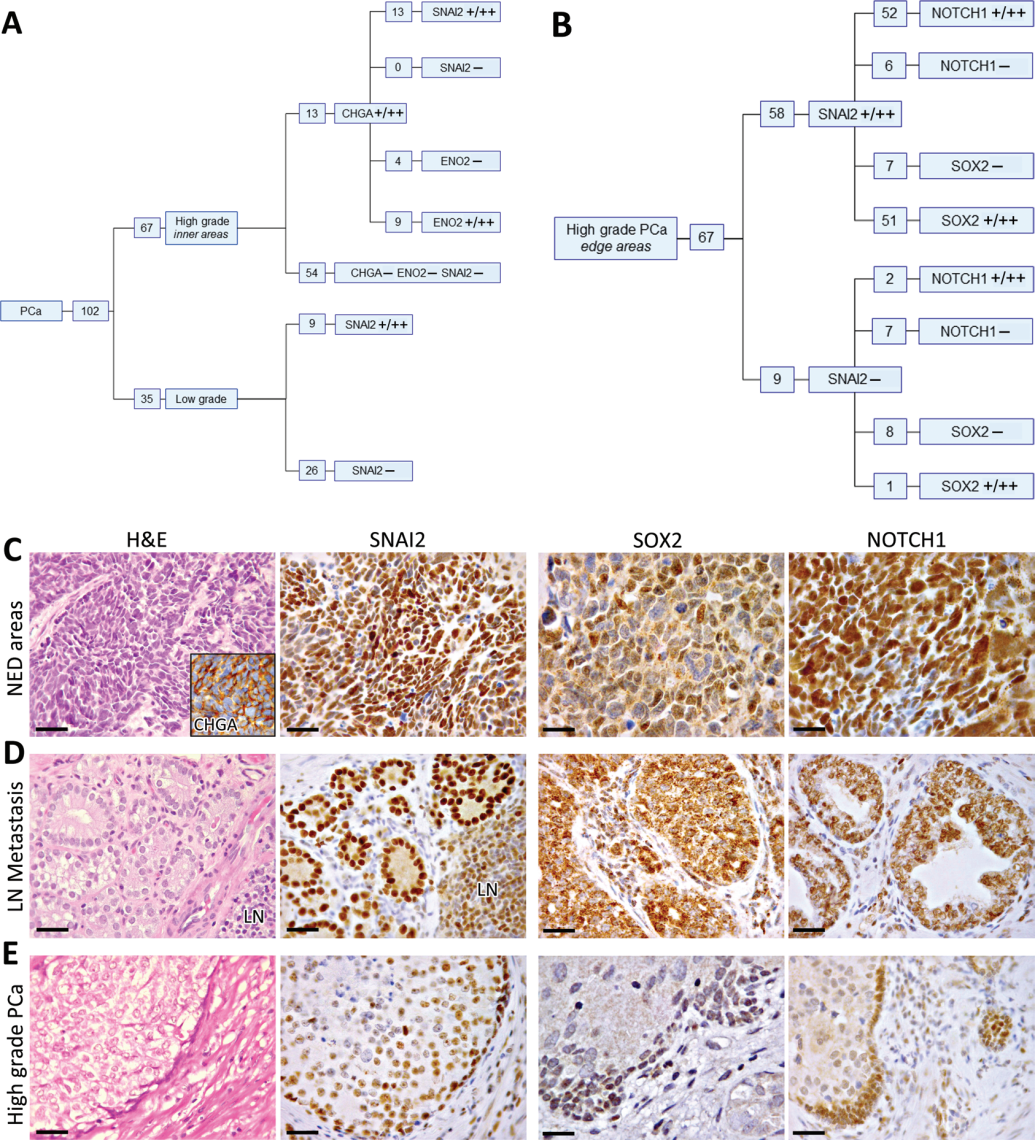
Immunohistochemical features of low- and high-grade foci in the primary PCa **(A) Distribution of SNAI2, CHGA and ENO2 expression in low-grade PCa, and in the inner areas of high-grade PCa.** A total positive correlation was disclosed by Spearman's rank correlation coefficient between the expression of SNAI2 and CHGA in clinical high-grade PCa samples, whereas a high correlation was detected between SNAI2 and ENO2 expression (ρ = 0.8, *p* < 0.0001). **(B) Distribution of SNAI2, NOTCH1 and SOX2 expression at the edge of high-grade PCa.** A positive relationship was disclosed, by Spearman's rank correlation coefficient, between the expression of SNAI2 and that of SOX2 (ρ = 0.63, *p* < 0.0001) and NOTCH1 (ρ = 0.58, *p* < 0.0001). **(C) Immunohistochemical features of NED areas developed in conventional adenocarcinomas.** Cancer cell nests forming CHGA+ (inset) NED areas firmly express SNAI2 (magnification X400) and SOX2, frequently NOTCH1 (magnification X630), with a distinct to strong staining. Scale bars: H&E and SNAI2 staining, 30 μm; SOX2 and NOTCH1 staining, 20 μm. **(D) Immunohistochemical features of lymph node metastases.** Lymph node (LN) metastases frequently express SNAI2, SOX2 and NOTCH1, with a distinct to strong staining. Magnification X400. Scale bars: 30 μm. **(E) Immunohistochemical features of high-grade PCa foci.** Cancer cell clusters at the edge of high-grade PCa frequently express SNAI2, SOX2, and NOTCH1. Magnification X400. Scale bars: 30 μm.

SNAI2 protein expression was truly heterogeneous insofar as it differed greatly from one focus to another and even within the same focus. In particular, a distinct to strong expression was confined to few cancer foci endowed with low (9/35) or high Gleason grade (13/67) (Figure 3A), and to cancer cell clusters bordering the expansion/invasion front of (58/67) high-grade poorly differentiated PCa foci (Figure 3B), whereas the inner groups of cells mostly stained slight to negative (Figure 1B and 3E).

To determine whether these clusters “escape” DNA methylation of the *SNAI2* gene promoter, detected in the majority of malignant epithelium, we carried out DNA sequencing in cells selectively microdissected from SNAI2 positive tumor edges versus those from the inner, SNAI2 negative, PCa foci with high Gleason grade. The *SNAI2* gene proximal promoter region was persistently unmethylated in cell clusters from the edge, whereas it was methylated in those from the inner foci (Figure 2C). Further evidence is thus provided of the role suggested for this epigenetic mechanism in the heterogeneous regulation of *SNAI2* gene expression.

The Chromogranin-A (CHGA) positive NED areas (13 PCa samples containing NED areas), which may develop in conventional adenocarcinomas (~10% frequency), especially within high-grade PCa (our observation and ref. 13), were marked by SNAI2 expression (Figure 3C), whose strength ranged from distinct to strong and involved nearly all cancer cells.

Moreover, immunohistochemical analysis of lymph node metastases in 15/102 PCa patients showed that they mostly (14/15) express SNAI2 with distinct to strong staining (Figure 3D).

Altogether, these observations lead us to assess whether knockdown of *SNAI2* in PCa cells may affect the expression of genes shaping the NED or metastatic potential of PCa cells.

### SNAI2 regulates the expression of NED markers, metastasis-suppressor and pluripotency genes

Since the *SNAI2* gene promoter was unmethylated in human PC3 cells, which clearly express SNAI2 mRNA, and protein at a level significantly (*p* < 0.05) higher than the 22Rv1 (~13 times), DU145 (~38 times) and LNCaP (~2 times) cell lines, we next performed, by specific small interfering RNA (siRNA), knockdown of *SNAI2* in the PC3 line.

**Figure 4:**
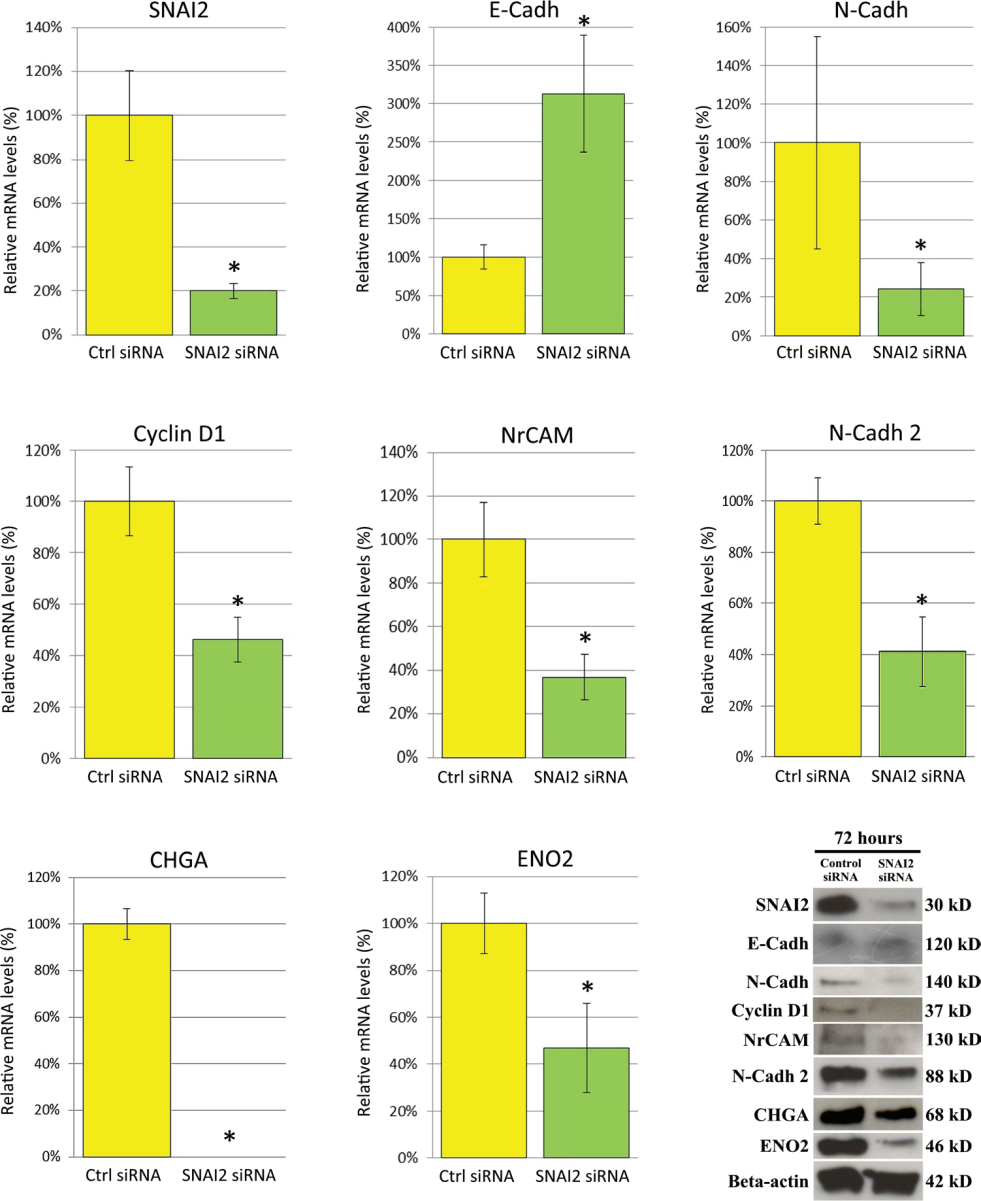
Effects of SNAI2-siRNA on the expression of genes associated to EMT, cell cycle, cell adhesion and neuroendocrine differentiation in PC3 cells, as assessed by real-time RT-PCR and Western Blot analysis Real-time RT-PCR showed that *SNAI2* mRNA expression in PC3 cells was silenced by 80% following 72 h of SNAI2-siRNA treatment. In *SNAI2*-silenced PC3 cells, *E-Cadh* was remarkably up-regulated, whereas *N-Cadh* was slightly, but significantly, down-regulated along with the *Cyclin D1*. The neural tissue associated adhesion molecules, *NrCAM* and *N-Cadh 2*, and the neuroendocrine markers, *CHGA* and *ENO2* were also considerably down-modulated in SNAI2-siRNA treated PC3 cells. Data are representative of three independent experiments. Western Blot analyses of Ctrl-siRNA and SNAI2-siRNA treated PC3 cells confirmed, at protein level, this gene expression regulation. β-actin was used as a loading control. *p < 0.05 Student's *t*-test compared with Ctrl cells.

Treatment of PC3 cells with SNAI2-siRNA resulted in a significant (*p* < 0.05) reduction in the expression of both *SNAI2* mRNA (up to 80%, *p* < 0.05), and protein (by 84% adjusted to β-actin) in comparison to Control (Ctrl)-siRNA treated cells, as determined, respectively by real-time RT-PCR and Western Blot analyses (Figure 4).

SNAI2-siRNA treatment did not affect expression of *SNAI1* or other EMT-regulating transcription factors, including Zinc finger E-box-binding homeobox 1 (*ZEB1*) and 2 (*ZEB2*), *TWIST1* and *TWIST2* (not shown).

*SNAI2* knockdown down-regulated *Cyclin D1* by 54%, with confirmation at the protein level, and modulated typical EMT cell surface markers such as E-Cadherin (*E-Cadh*), which was up-regulated (213%) and N-Cadherin (*N-Cadh*), which was slightly, but significantly (*p* < 0.05) down-regulated at the transcriptional (76%) and protein level (Figure 4).

The neural tissue associated adhesion molecules, namely neuronal cell adhesion molecule (*NrCAM*) and N-Cadherin 2 (*N-Cadh 2*), were dramatically down-regulated by 63% and 59% respectively at the transcriptional level with confirmation at the protein level (Figure 4), whereas the expression of neural cell adhesion molecule L1 (*L1CAM*) remained unaltered.

Key genes for NED were clearly altered by SNAI2-siRNA treatment since the expression of *CHGA* was completely abolished and that of Enolase 2 (*ENO2*) significantly (*p* < 0.05) decreased, as revealed by real-time RT-PCR analyses (53%), and confirmed at protein level by Western Blotting (Figure 4), while the expression of synaptophysin (*SYP*) and neurogenic differentiation factor 1 (*NeuroD1*) only showed a trend towards down-regulation (not shown).

To assess whether SNAI2 may affect PCa cell malignancy by regulating other gene pathways besides those tightly associated with EMT, we next investigated the expression, in *SNAI2* knockdown PC3 cells, of a set of metastasis-suppressor [17, 18] and pluripotency genes which have been associated with cancer aggressiveness and metastatic potential [11, 12].

**Figure 5:**
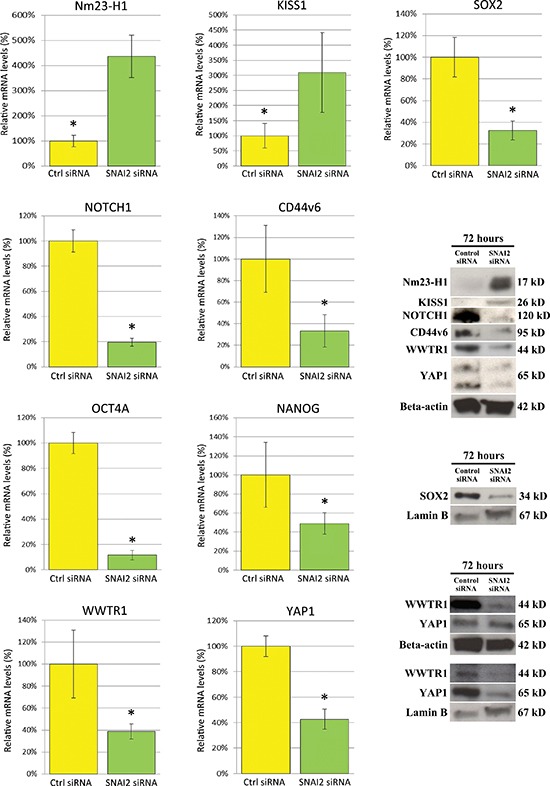
Effects of SNAI2-siRNA on the expression of metastasization- and pluripotency-associated genes in PC3 cells, as assessed by real-time RT-PCR and Western Blot analysis Expression of *Nm23-H1* and *KISS1* was considerably up-regulated in PC3 cells after SNAI2-siRNA treatment. By contrast, the stemness-related genes *CD44v6*, *SOX2*, *NOTCH1*, along with *WWTR1/TAZ*, *YAP1*, *OCT4A* and *NANOG* were significantly down-regulated. Data are representative of three independent experiments. Western Blot analysis of Ctrl-siRNA and SNAI2-siRNA treated PC3 cells confirmed, at protein level, this gene expression regulation for only the first five genes, with β-actin or Lamin B (evaluation of the nuclear protein fractions of SOX2, WWTR1/TAZ and YAP1) as a loading control. *p < 0.05 Student's *t*-test compared with Ctrl cells.

Among the metastasis-suppressor genes, both *Nm23-H1* and *KISS1* were significantly (*p* < 0.05) up-regulated in *SNAI2* knockdown PC3 cells, respectively by 337% and 209% at the transcriptional level, with confirmation at the protein level (Figure 5), whereas the expression of *CD82/KAI-1*, phosphatidylethanolamine-binding protein 1/Raf kinase inhibitor protein (*PEBP1/RKIP*), dual specificity mitogen-activated protein kinase 4 (*MAP2K4*) and 7 (*MAP2K7*) remained substantially unchanged.

*SNAI2* knockdown also resulted in significant (*p* < 0.05) down-regulation of the expression levels of pluripotency and self-renewal associated genes such as *SOX2* by 68%, neurogenic locus notch homolog protein 1 (*NOTCH1*) by 80%, *CD44v6* by 67% (Figure 5), octamer-binding transcription factor 4A (*OCT4A*) by 88%, and *NANOG* by 51%, as assessed by real-time RT-PCR. These down-regulations were distinctly confirmed at the protein level for only the first three genes (Figure 5).

Expression levels of *SOX9*, *BMI1*, krueppel-like factor 4 (*KLF4*), *C-MYC* and sonic hedgehog (*SHH*) remained substantially unaltered.

Interestingly, the transcriptional regulators WW domain-containing transcription regulator protein 1/transcriptional coactivator with PDZ-binding motif (*WWTR1/TAZ*) and Yes-associated protein 1 (*YAP1*), known to be involved in the self-renewal and metastatic properties of cancer cells [19, 20], were significantly (*p* < 0.05) down-regulated in SNAI2-siRNA treated cells in comparison with control cells, at the transcriptional level by 61% and 57% respectively, with confirmation at the protein level (Figure 5). Furthermore, distinct assessment of the nuclear (with Lamin B as a loading control) and cytoplasmic fractions of both TAZ and YAP1 proteins revealed that TAZ expression was down-regulated in both compartments and YAP1 substantially in the nuclei (Figure 5, bottom right WB images).

To confirm the finding of SNAI2-dependent regulation of genes governing NED, metastasization, and stemness, we finally transfected the *SNAI2* gene into the DU145 cell line, which had revealed the lowest transcription level of endogenous *SNAI2*, and assessed expression levels of the above reported genes in *SNAI2* over-expressing cells.

**Figure 6:**
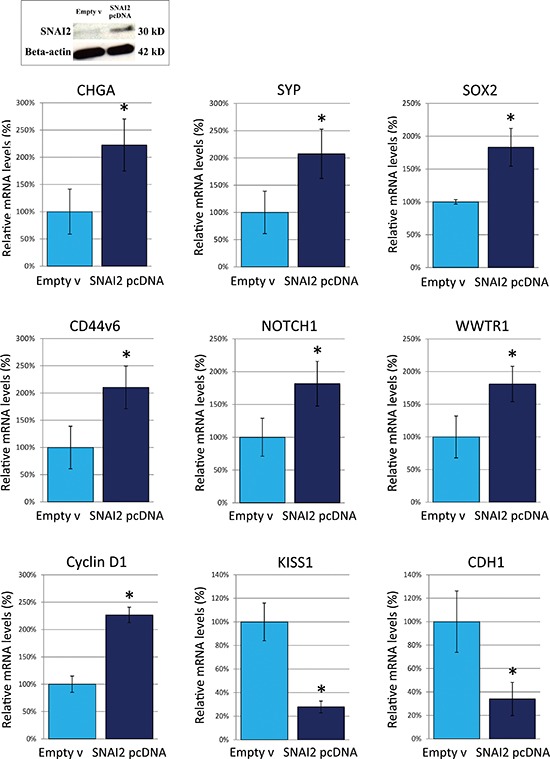
Effects of *SNAI2*-gene transfection on the expression of neuroendocrine differentiation-, metastasization- and pluripotency-associated genes in DU145 cells Western Blot analyses showed that, in *SNAI2*-gene transfected DU145 cells, SNAI2 protein expression was up-regulated by 259% (adjusted to β-actin). Real-time RT-PCR showed that *CHGA* and *SYP* were up-regulated by 222%, and 208%, respectively. Within the stemness-related genes, the expression levels of *SOX2*, *CD44v6* and *NOTCH1*, along with that of *WWTR1/TAZ*, increased by 183%, 210%, 182%, and 181%, respectively. *Cyclin D1* was up-regulated by 227%, whereas *KISS1* and *CDH1* were down-regulated by 72%, and 66%, respectively. *p < 0.05 Student's *t*-test compared with Ctrl cells.

DU145 cell transfection with *SNAI2* expressing vector resulted in a significant (*p* < 0.05) increase in the expression of both *SNAI2* mRNA (more than 90%, *p* < 0.05), and protein (by 259% adjusted to β-actin) in comparison with Ctrl-empty vector transfected cells, as determined, respectively by real-time RT-PCR and Western Blot analyses (Figure 6). Within the NED-related genes, *CHGA* and *SYP* were up-regulated (*p* < 0.05) by 222%, and 208% respectively (Figure 6), starting 24 h after DU145 cell transfection, whereas *ENO2* only displayed a trend towards up-regulation (not shown). Within the stemness-related genes, the expression levels of *SOX2*, *NOTCH1*, and *CD44v6*, along with that of *WWTR1/TAZ*, were significantly (*p* < 0.05) increased (by 183%, 182%, 210%, and 181%, respectively) in SNAI2-over-expressing cells. A significant up-regulation of *Cyclin D1* (by 227%) was evidenced 48 h after cell transfection, whereas 24 h later, a significant (*p* < 0.05) down-regulation of the metastasis-suppressor gene *KISS1*, by 72%, and of *CDH1*, by 66%, also occurred (Figure 6).

### Expression of *SNAI2* in clinical PCa samples was associated with that of the stemness-related transcription factors *SOX2* and *NOTCH1* and, with that of the NED-related genes *CHGA* and *ENO2*

Based on the evidence that *SNAI2* silencing may substantially down-regulate *SOX2* and *NOTCH1* expression, we immunohistochemically analysed patients' prostate tissue to assess the relationship between the expression of *SNAI2* and that of these two down-stream target genes.

**Figure 7:**
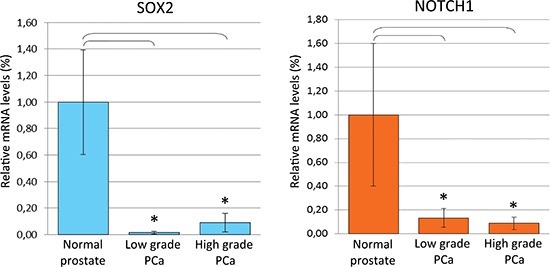
SOX2 and NOTCH1 mRNA expression in normal and cancerous prostate epithelium from PCa patients **(A)** Histogram representing the relative expression ± SD of *SOX2* mRNA in microdissected histologically normal epithelium and its neoplastic counterparts with low or high Gleason grades, from prostatectomized patients (groups of 15), normalized with the housekeeping gene HPRT. One-way ANOVA for comparisons between epithelial compartments of normal prostate, low- and high-grade PCa: *p* < 0.0001. *p < 0.01 Tukey HSD Test compared with normal prostate epithelium. **(B)** Histogram representing the relative expression ± SD of *NOTCH1* mRNA in microdissected histologically normal epithelium and its neoplastic counterparts with low or high Gleason grades, from prostatectomized patients (groups of 15), normalized with the housekeeping gene HPRT. One-way ANOVA for comparisons between epithelial compartments of normal prostate, low- and high-grade PCa: *p* < 0.0001. *p < 0.01 Tukey HSD Test compared with normal prostate epithelium.

Real-time RT-PCR analyses of microdissected normal and neoplastic epithelium revealed that the mean levels of *SOX2* and *NOTCH1* mRNA were considerably (*p* < 0.05) down-regulated in the neoplastic epithelial cell populations from both low- (*SOX2*: 65 times, *NOTCH1*: 7,5 times) and high- (11 times for both genes) grade PCa (with no appreciable difference between them) in comparison with the normal epithelium (Figure 7). Fluctuations of *SOX2* and *NOTCH1* expression from one sample to another were associated with those of *SNAI2* (*r* = 0.69 for *SOX2*; *r* = 0.51 for *NOTCH1*), resulting in a mean ± SD of *SOX2* and *NOTCH1* expression levels that paralleled, in the epithelium, *SNAI2* expression levels.

Furthermore, in accordance with the heterogeneity of SNAI2 expression in the primary PCa, the strength of the staining of both SOX2 and NOTCH1 was frequently distinct or strong in the tumor cell clusters forming the expansion/invasion front of high-grade PCa foci (Figure 3B and 3E).

In particular, SNAI2 staining was shown at the edge of 58/67 high-grade PCa foci. 51 out of 58 SNAI2 positive tumor edges also stained for SOX2 and 52/58 also stained for NOTCH1 (Figure 3B). Concomitant expression of all three markers was found at the edge of 50/67 high-grade PCa foci (Figure 3B). Spearman's rank correlation coefficient disclosed a positive relationship between the expression of SNAI2 and those of SOX2 (ρ = 0.63, *p* < 0.0001) and NOTCH1 (ρ = 0.58, *p* < 0.0001).

Furthermore, immunohistochemical analysis of lymph node metastases revealed distinct to strong SOX2 and NOTCH1 expression in 11/15 and 12/15 metastatic lesions, respectively (Figure 3D).

Lastly, all the 13 NED areas in our sample collection were marked by distinct to strong SOX2 expression (Figure 3C), and 11/13 also showed a distinct to strong NOTCH1 expression, involving nearly all cancer cells (Figure 3C).

As to the relationship between *SNAI2* and NED-related gene expression, a total positive correlation emerged between the expression of SNAI2 and CHGA in clinical high-grade PCa samples, since all the 13 CHGA-positive NED areas also expressed SNAI2, whereas the 54 CHGA-negative high-grade PCa lacked SNAI2 expression (apart from the tumor burden) (Figure 3A and 3C). In addition, a high correlation, was detected between SNAI2 and ENO2 expression, ρ = 0.8, *p* < 0.0001, since a distinct to strong ENO2 expression involved 9/13 NED areas, whereas it was absent in the 54 SNAI2-negative high-grade PCa (Figure 3A).

## DISCUSSION

This study sheds new light on the role of the *SNAI2* gene in the pathogenesis and progression of PCa in function of the heterogeneous nature of this disease. Here we report, for the first time, that PCa is marked by the transcriptional silencing of *SNAI2* in most of the neoplastic epithelium, irrespective of its differentiation grade, in association with gene promoter methylation. This epigenetic silencing appears to be quite selective since it does not involve NED areas and invasion fronts of high-grade tumors. The basal cell loss distinctive of malignant prostatic glands [15] is not responsible for the loss of *SNAI2* expression in PCa, as shown by the comparable levels of *SNAI2* transcript within basal cells, more differentiated luminal secretory cells, and the whole glandular epithelium, which also means that, in the normal prostatic epithelium, *SNAI2* expression is unrelated to the different self-renewal capabilities of the cell compartments.

The finding of a substantial *SNAI2* down-modulation in neoplastic as compared to normal prostate epithelia agrees with data obtained by gene expression profiling of PCa samples, by Urbanucci *et al.* [21], and of microdissected PCa epithelia, by Tomlins *et al* [22]. A similar unexpected finding has recently been described in melanoma development [23, 24] suggesting that SNAI2's role in the pathogenesis of cancer goes beyond its well-known EMT regulation. Our data reveal that, in addition to the canonical cadherin switch, represented by the opposite E-Cadh/N-Cadh expression, and Cyclin D1 regulation, which confirms its influence on the cell cycle [25], SNAI2 heavily affects the expression of the neural tissue associated adhesion molecules N-Cadh 2 and Nr-CAM both critically involved in cell migration and invasion and strongly down-regulated in PCa cells following *SNAI2* silencing.

Above all, our study shows that SNAI2 may condition prostate carcinogenesis as an up-stream regulator of multiple cancer progression pathways governed by pluripotency-, neuroendocrine- or metastasization-related gene. It also lends support to recent efforts to develop SNAI2-targeting drugs [26, 27].

*SNAI2* gene dramatically modulates three key regulators of the cell stem state, associated with invasion and metastasization, namely CD44v6 [28, 29], SOX2 [30–34] and NOTCH1 [35–38], as revealed by both *SNAI2* silencing and over-expression experiments, in PCa cells *in vitro* and, probably, in PCa tissue *in vivo*. The topographic distribution of SOX2 and NOTCH1 expression, in fact, is closely related to that of SNAI2, thus strengthening the idea that, as downstream targets, their expression may be boosted by SNAI2 in selected PCa areas, namely; **a**. cell clusters at the invasive front of poorly differentiated, high-grade, PCa foci, **b**. NED areas developed in their context, and **c**. lymph node metastases. The frequency of their concomitant expression in these sites strongly suggests their cooperation in promoting PCa progression.

As emerging regulators of cancer stemness, growth and malignant progression, the two closely related transcriptional regulators YAP1 and TAZ are drastically down-modulated by *SNAI2* gene silencing, whereas TAZ in particular is also up-regulated by *SNAI2* over-expression. TAZ is required to sustain self-renewal and tumor-initiation capacities in breast cancer stem cells [20, 39]. It is turned on by over-expression of the EMT-inducing transcription factors TWIST or Snail [20], and is required for TWIST-induced self-renewal. TAZ itself can sustain EMT [20, 40]. Nuclear localization promotes TAZ/YAP1 transcriptional activity driving cell proliferation, transformation and tumorigenicity [41]. Our present demonstration of a significant decrease in the nuclear fraction of TAZ and particularly YAP1 proteins following *SNAI2* silencing suggests that SNAI2 regulates not only their expression, but also their translocation and transcriptional activation. The TAZ/YAP1 system may thus be proposed as a novel key downstream effector of SNAI2's function in PCa. Similarly, the metastasis-suppressors Nm23-H1 and, particularly, KISS1 have been revealed as downstream effectors of SNAI2's pro-metastatic role, since expression of both greatly increases following *SNAI2* knockdown, whereas *SNAI2* over-expression substantially down-regulates the *KISS1* gene expression only. The mechanisms whereby Nm23-H1 and KISS1 exert metastasis-suppressor functions involve multiple pathways and are not completely clear [42, 43]. Their reduced expression, however, is associated with increased cancer cell invasiveness, metastasization and a poorer clinical outcome in most tumor types, including PCa, while their over-expression hinders cancer cell migration and invasion [44, 45].

As a hallmark of PCa aggressiveness, NED has been associated with tumor progression and development of castration-resistant disease [13, 14]. Normal prostatic neuroendocrine cells are thought to be derived from the neural crest during embryogenesis [46]. SNAI2 drives their migration [4], and has been described as a neural crest and neural plate border marker [47, 48]. We show that *SNAI2* gene silencing abolishes the expression of *CHGA* and dramatically reduces that of *ENO2*, whereas its over-expression substantially up-regulates *CHGA* and *SYP* expression. These data are in line with the finding of a tight correlation between expression of NED markers and that of SNAI2 in the clinical samples, and suggest that firm and strong SNAI2 expression promotes NED within high-grade PCa, thus favoring tumor progression along these pathways [14]. Our present assessment of the involvement of SNAI2 in a variety of malignancy-related gene pathways, has not disclosed a complete SNAI2-associated gene expression profile in the clinical samples, achievable by DNA microarray or next generation sequencing, and its correlation with the follow-up. This limitation may be overcome in the event of a future substantial increase in patient recruitment and biological sample collection.

Taken as a whole our findings provide novel insight into the complex molecular heterogeneity of PCa, and lead to the following conclusions:

I. An aberrant and selective regulation of SNAI2 expression marks the growth and progression of PCa, since it is lacking in the former and clearly present in the latter.

II. Silencing of *SNAI2* in most PCa epithelia may turn off the expression of NED markers and pluripotency genes while turning on that of specific metastasis-suppressor genes.

III. Selective SNAI2 expression in cancer cell clusters at the invasion front, or in NED areas of poorly differentiated PCa may endow these cells with stemness and/or neuroendocrine traits and migration/invasion property, thus promoting their self-renewal and metastatic capabilities.

These conclusions strongly identify the *SNAI2* gene as a key target for modern strategies to prevent or cure the metastatic disease.

## MATERIALS AND METHODS

### Ethics statement

Investigation has been conducted in accordance with the ethical standards and according to the Declaration of Helsinki and according to national and international guidelines and has been approved by the authors' institutional review board.

### Patients and samples

We collected tissue samples, and the clinical and pathological data of 102 patients aged 58–73 treated by radical prostatectomy for PCa.

PCa samples were graded [49] as Gleason score 5 (*n* = 18), 6 (*n* = 14), 7 (*n* = 32), 8 (*n* = 27), and 9 (*n* = 11) and staged [50] as pT2, organ-confined cancer (*n* = 58 [13 T2aN0M0, 22 T2bN0M0, 18 T2cN0M0, and 5 T2cN1M0]) and pT3, capsular penetration (*n* = 44 [19 T3aN0M0, 4 T3aN1M0, 15 T3bN0M0, and 6 T3bN1M0]). On the bases of pathological TNM classification, the cases were divided into: 53 in Stage II, 34 in Stage III and 15 in Stage IV (with metastases to the pelvic lymph nodes). In addition, we obtained normal prostates (histologically negative for PCa or benign prostatic hyperplasia) from 16 untreated patients, aged 55–64, prostatectomized for bladder cancer (control patients). Sample collection and processing are described in the online Supporting Information.

Written informed consent was obtained from patients. The study has been approved by the Ethical Committee of the “G. d'Annunzio” University of Chieti (Italy), and Local Health Authority No. 2 in PROT 1945/09 COET of 14/07/2009, and performed in accordance with the principles outlined in the Declaration of Helsinki.

### Laser capture microdissection

For LCM, 10 μm frozen sections (two sections per sample) from cancer and normal prostate specimens (of both control and PCa patients) were analyzed.

Neoplastic foci with low Gleason grade were obtained from 35/102 PCa patients, whereas those with high grade were obtained from the other 67/102 PCa patients.

Epithelial and stromal components were dissected from cancer and normal prostate specimens as described in the online Supporting Information.

### Methylation analysis by bisulfite genomic sequencing

Genomic DNA was extracted from microdissected cells using the QIAamp DNA Micro Kit and then bisulfite-converted with the EpiTect Bisulfite Conversion Kit (both from Qiagen, Hilden, D).

The sequence reaction was performed with BigDye® Terminator v3.1 Cycle Sequencing Kit (Applied Biosystems, Foster City, CA, USA) with the primers used for the PCR reaction.

The sequence products were separated on a fluorescent capillary sequencer (ABI 3130xl, Applied Biosystems) and analyzed with SeqScape software (Applied Biosystems). Details on the sequence reaction and the primers used are described in the online Supporting Information.

### Immunohistochemistry

Single and double Immunohistochemistry on formalin-fixed, paraffin-embedded samples was performed as described in the online Supporting Information.

### The Morphometric analyses

SNAI2, CHGA, ENO2, SOX2 and NOTCH1 expression by primary tumors or lymph node metastases was evaluated using the following criteria based on 1) the widening of the staining expressed as the percentage of tumor or metastasis stained i.e.: < 50%, ≥ 50% ≤ 70%, and > 70%, and 2) the strength of the staining: defined as negative (0), slight (1), moderate (2) or intense (3).

Thus, immunostaining was defined as:

***strong*** ++, when a) the widening was > 70% and its strength range slight (1) to intense (3), or b) the widening was > 50% ≤ 70% and its strength range moderate (2) to intense (3);

***distinct*** +, when a) the widening was > 50% ≤ 70% and its strength was slight (1) or b) the widening was = 50% and its strength range slight (1) to intense (3);

***absent*** −, when the widening was < 50% and its strength was slight (1) to negative (0).

Results were expressed as the percentage of positive cells, or as the mean ± SD of positive cells/field, evaluated by light microscopy on single immunostained formalin-fixed paraffin-embedded sections at X400 in a 85431.59 μm^2^ field.

Six to 10 high-power fields (depending on the tumor width) were examined per section and three sections per sample were evaluated.

Stained sections were examined by two pathologists (EDC and CS) in a blind fashion, with very good agreement (κ value = 0.82) [51].

### Cell cultures, *SNAI2* silencing and over-expression

The human PCa cell lines 22Rv1, DU145, LNCaP (from the American Type Culture Collection, Manassas, VA, USA) and PC3 (Interlab Cell Line Collection, CBA/IST San Martino, Genova Italy), were cultured in RPMI 1640 with 10% FCS. Cell lines were obtained directly from the above mentioned cell banks that performed cell line characterizations by Short Tandem Repeats (STR) profile analysis. PC3, 22Rv1, DU145 and LNCaP were passaged in our laboratory for fewer than 6 months after resuscitation.

RNA was extracted using RNeasy Mini Kit (Qiagen) and subjected to real-time RT-PCR, to detect *SNAI2* mRNA expression levels, by the RT^2^ First Strand cDNA Synthesis kit (SABioscience, Frederick, MD, USA).

Silencing of human *SNAI2* was achieved by transfection of 1 × 10^6^ PC3 cells with lipofectamine RNAiMax reagent (Life Technologies, Carlsbad, CA, USA) using 3 μg of Mission esiRNA SNAI2 or irrelevant siRNA Universal Negative Control (Sigma-Aldrich, St. Louis, MO, USA).

RNA was extracted after 72 hours and subjected to real-time RT-PCR. Furthermore, cell pellets from PC3 cells were collected for Western Blotting.

For overexpression of *SNAI2* in DU145 cells, the GFP-tagged *SNAI2* expression vector or empty vector (pCMV6-AC-GFP) (both from OriGene Technologies, Rockville, MD, USA) was transfected into 1 × 10^6^ cells using lipofectamine RNAiMax reagent (Life Technologies).

Twenty-four, 48, and 72 hours after DU145 cell transfection, RNA was extracted and subjected to real-time RT-PCR, and cell pellets were collected for Western Blotting.

### Real-time RT-PCR

The real-time RT-PCR was performed on RNA extracted from microdissected cells and *SNAI2*-silenced and Ctrl cells, using the Quantitect Reverse Transcription Kit for the reverse transcription and the QuantiFast SYBR Green PCR Kit for real-time PCR (both from Qiagen). The real-time RT-PCR was done, with primers listed in the online Supporting Information using the MiniOpticon System (Bio-Rad, Hercules, CA, USA).

### Western blotting

Western blot was performed using total proteins and nuclear extracts obtained from approximately 2.0 × 10^6^ cells. Cells were collected following standard procedures. Then, 1.0 mL of ice cold RIPA Lysis buffer (Thermo Scientific, Waltham, MA, USA) was added, with freshly added Protease and Phosphatase Inhibitors Cocktails (Thermo Scientific). SOX2, WWTR1/TAZ and YAP1 were investigated in nuclear fractions, obtained using CelLytic™ NuCLEAR™ EXTRACTION KIT (Sigma-Aldrich). Total proteins and nuclear fractions were measured in the extract by the Bradford assay.

The nuclear fractions and the whole cell lysates were examined on Mini-PROTEAN TGX Gels 4–20% (Bio-Rad). Proteins were transferred from gels on Immun-Blot PVDF Membranes (Bio-Rad) in the transfer buffer (glycine, tris [pH 8.4] and methanol) using Mini Trans-Blot Cell apparatus (Bio-Rad). Membranes containing proteins were blocked with milk 5X (Sigma-Aldrich) in TBST and, subsequently, probed with primary and horseradish peroxidase conjugated secondary antibodies following standard procedures. Proteins transferred membranes were washed with TBST and developed with Pierce ECL Western Blotting Substrate (Thermo Scientific). Details are provided in the online Supporting Information.

### Statistical analysis

Between-group differences in the relative expression of *SNAI2*, *SOX2* or *NOTCH1*, by real-time RT-PCR, in epithelial or stromal compartments of normal and neoplastic prostate tissues were assessed by ANOVA. The difference between each pair of means was evaluated with the Tukey HSD test. Differences in the relative gene expression, by real-time RT-PCR, between SNAI2-silenced, SNAI2-overexpressing and Ctrl cells were assessed by Student's *t*-test. The χ2 test and the Mann–Whitney U probability test were used to examine the association between *SNAI2* expression levels and the clinical and pathologic characteristics. The Spearman's rank correlation coefficient (ρ) was used to examine the correlation between SNAI2 protein expression and immunohistochemical staining for SOX2, NOTCH1, CHGA, and ENO2 whereas the Pearson product-moment correlation coefficient (*r*) was used to examine the correlation between mRNA expression levels of *SOX2* or *NOTCH1* and *SNAI2*. The SPSS software, version 11.0 (IBM, Armonk, NY, USA) was employed, with *p* < 0.05 as the significance cut-off.

## SUPPLEMENTARY DATA TABLES


