# Supplementary material for: SNAI2/Slug gene is silenced in prostate cancer and regulates neuroendocrine differentiation, metastasis-suppressor and pluripotency gene expression
Source: Oncotarget. 2015 Feb 18;6(19):17121–34. doi: 10.18632/oncotarget.2736 (PMC4627296; doi:10.18632/oncotarget.2736)
Supplement: Supplementary file 1 [file oncotarget-06-17121-s001.pdf]

SUPPLEMENTARY DATA

MATERIALS AND METHODS

Patients and samples

Patients entering the study had not received hormone or immunosuppressive treatments or radiotherapy, and were free from immune system diseases. Clinical and pathological stages were determined according to the 7th edition of the TNM Classification of Malignant Tumors. Tumor grade was assessed according to the Gleason scoring system from the prostate biopsies.

The biological samples were cancer and normal prostate specimens (formalin-fixed and frozen) and draining lymph nodes (formalin-fixed) from PCa patient who underwent radical prostatectomy between 2009 and 2013 at the "S.S. Annunziata" Hospital, Chieti, Italy. One-half of each tissue sample was fixed in 4% formalin and embedded in paraffin. The other was embedded in Killik frozen section medium (Bio-Optica, Milano, Italy), snap frozen in liquid nitrogen, and preserved at –80°C. For histology, paraffin-embedded samples were sectioned at 4 μm and stained with H&E. Single and double immunohistochemistry were done on paraffin-embedded or frozen sections, depending on the antibody (Ab) used.

Laser capture microdissection (LCM)

LCM was performed using the P.A.L.M. Micro Beam System (P.A.L.M. Microlaser Technologies, Bernried, D). For LCM, 10 μm frozen sections from cancer and normal prostate specimens (of both control and PCa patients) were mounted on polyethylene naphthalate membrane-covered slides (P.A.L.M. Microlaser Technologies), thawed at room temperature, and immersed in cold acetone (5 min). Immediately after H&E staining, sections were used for LCM. From 1000 to 1500 selected cells were cut and catapulted intact into the cap of an LPC-Microfuge Tube (P.A.L.M.), and RNA was immediately isolated with the RNeasy Plus Micro kit (Qiagen, Hilden, D). Two sections per sample were analyzed.

Epithelial and stromal components were dissected from cancer and normal prostate specimens (of both control and PCa patients) with discrimination between neoplastic foci with low (well differentiated) *versus* high (poorly differentiated) Gleason grade (≤ 3 *versus* > 3). Foci with the same grade were microdissected from each specimen.

Foci with low grades were obtained from 35/102 PCa patients, whereas foci with high grades were obtained from the other 67/102 PCa patients. The stroma was isolated among the glands of both low-grade and high-grade PCa, or in the histologically normal zones far from the PCa.

All reagents used for LCM were prepared with Ultrapure DNase/RNase-free distilled water (Invitrogen, Paisley, UK).

Methylation analysis by bisulfite genomic sequencing

Bisulfite-sequencing PCR primers, *SNAI2* Forward (5′-TTGGTTTAAAATGGGTTGTTT-3′) and *SNAI2* Reverse (5′-TATACAAAACAAACCTCTCCAAAT-3′) were designed, using the Methyl Primer Express program (Applied Biosystems, Foster City, CA, USA), for specific amplification of successfully converted DNA strands only and did not contain any CpG sites within their sequences. They were synthesized by the Sigma-Aldrich Corporation (St. Louis, MO, USA) and used for amplification of a 309 bp region of the *SNAI2* gene promoter, located 130 bp upstream from the transcription start site and containing 9 sparsely spaced CpGs in its central portion. Thermal reactions were performed on a MJ Mini Gradient Thermal Cycler (Bio-Rad, Hercules, CA, USA). PCR conditions were as follows: 95°C for 2 min, 44 cycles of 95°C for 1 min., 55°C for 30 sec.,72°C for 1 min., followed by 72°C for 7 min. The PCR was carried out with the AmpliTaq Gold® 360 Master Mix (Applied Biosystems). The sequence reaction was performed with the primers used for the PCR reaction. Cycle sequencing conditions were as follows: 96°C for 1 min., 25 cycles of 96°C for 10 sec., 55°C for 5 sec., 60°C for 4 min.

Immunohistochemistry

Immunohistochemistry on formalin-fixed, paraffin-embedded samples was performed with the following primary Abs: anti-AMACR (clone 13H4), anti-Chromogranin A/CHGA (clone DAK-A3), anti-Cytokeratin 5.6 (clone D5/16 B4) (all from Dako, Glostrup, DK); anti-Cytokeratin 18 (clone DC-10, Leica Biosystems, Newcastle Upon Tyne, UK); anti-NOTCH1 (clone 3E12, Novus Biologicals, Cambridge, UK); anti-SNAI2 (clone 1A6, Origene Technologies, Rockville, MD, USA); anti-SOX2 (clone 57CT23.3.4, Abcam, Cambridge, UK). Sections were treated with H_2O_2/3% for 5 min to inhibit endogenous peroxidase and then washed in H_2O. Antigen was unmasked with heat-induced epitope retrieval in ethylenediaminetetracetic acid (EDTA) buffer at pH 8 (prior to incubation with SNAI2, SOX2 and NOTCH1 Abs). The slices were then held for 20 min at room temperature. After washing in PBS/Tween-20, sections were incubated for 30 min with the primary Ab and immunocomplexes were detected using the Bond Polymer Refine Detection kit (Leica Biosystems) according to the manufacturer's protocol. Negative controls were formed by replacing the primary Ab with 10% non-immune serum. Further controls were obtained by omitting the secondary Ab. Controls were always negative.

For SNAI2/AMACR, SNAI2/Cytokeratin 5.6 and SNAI2/Cytokeratin 18 double stainings on formalin-fixed paraffin-embedded samples, sections were deparaffinized,

treated with H_2O_2/3% for 5 min to inhibit endogenous peroxidase, and then washed in H_2O. The slices were then incubated for 30 min with the first primary antibody (Ab) (mouse anti-SNAI2 Ab) followed by detection with the Bond Polymer Refine Detection Kit (Leica Biosystems) according to the manufacturer's protocol. Then, sections were incubated for 30 min with the second primary Ab (rabbit anti-AMACR, mouse anti-Cytokeratin 5.6, mouse anti-Cytokeratin 18) followed by detection with the Bond Polymer Refine Red Detection Kit (Leica Biosystems) according to the manufacturer's protocol.

Real-time RT-PCR

The primers for *CD44v6, CD82/KAI1, KISS1, Nm23-H1, MAP2K4, MAP2K7, NANOG, OCT4A, PEBP1/RKIP, SNAI1, SNAI2, SOX2, TWIST1, TWIST2, YAP1, ZEB1, ZEB2,* and the housekeeping gene hypoxanthine phosphoribosyltransferase 1 (*HPRT*) were designed and synthesized by Sigma-Aldrich Corporation: *CD44v6* forward 5′-AGGAACAGTGGTTTGGCAAC-3′ and *CD44v6* reverse 5′-CGAATGGGAGTCTTCTCTGG-3′; *CD82/KAI1* forward 5′- AGCAGTTTCATCTCTGTCCT-3′ and *CD82/KAI1* reverse 5′-GCGTTGTCTGTCCAGTTG-3′; *KISS1* forward 5′-GAACTACAACTGGAACTCCTT-3′ and *KISS1* reverse 5′-ATGCTCTGACTCCTTTGG-3′; *Nm23-H1* forward 5′-GTGAGTTCTCCCTGTACA-3′ and *Nm23-H1* reverse 5′-AGCAATGCAACAATATGAAGTA-3′; *MAP2K4* forward 5′-ACGCAAAGCACTGAAGTT-3′ and *MAP2K4* reverse 5′-AGTCTCTCTATGTGTGGGTTT-3′; *MAP2K7* forward 5′-GTTCATCACCAACACGGA-3′ and *MAP2K7* reverse 5′-CAATCGCCACTGTCATCT-3′; *NANOG* forward 5′-TCTTCCACCAGTCCCAAA-3′ and *NANOG* reverse 5′-GCGTCACACCATTGCTAT-3′; *PEBP1/RKIP* forward 5′-ACTCACTCACTCTGATTTATGTTT-3′ and *PEBP1/RKIP* reverse 5′-TGTGTACCATTCTGGACTCA-3′; *OCT4A* forward 5′-CCCCTGGTGCCGTGAA-3′ and *OCT4A* reverse 5′-GCAAATTGCTCGAGTTCTTTCTG-3′; *SNAI1* forward 5′-CCTCTTCCTCTCCATACCT-3′ and *SNAI1* reverse 5′-TTCATCAAAGTCCTGTGGG-3′; *SNAI2* forward 5′-TGTCATACCACAACCAGAGA-3′ and *SNAI2* reverse 5′-CTTGGAGGAGGTGTCAGAT-3′; *SOX2* forward 5′- AGAGAGAAAGAAAGGGAGAGA-3′ and *SOX2* reverse 5′-AATCAGGCGAAGAATAATTTGG-3′; *TWIST1* forward 5′-CGGAGACCTAGATGTCATT-3′ and *TWIST1* reverse 5′-CTGTCTCGCTTTCTCTTTT-3′; *TWIST2* forward 5′- AACTGGACCAAGGCTCTC -3′ and *TWIST2* reverse 5′-GCGGCGTGAAAGTAAGAAT-3′; *YAP1* forward 5′-TTCCTCTCCAGCTTCTCTGC-3′ and *YAP1* reverse 5′-GATGCTGAGCTGTGGGTGTA-3′; *ZEB1* forward 5′-CCAACAGACCAGACAGTG-3′ and *ZEB1* reverse 5′-TGACTCGCATTCATCATCTT-3′; *ZEB2* forward 5′-CGGAGACTTCAAGGTATAATCTATC-3′ and *ZEB2* reverse 5′-GTTACGCCTCTTCTAATGACAT-3′; *HPRT* forward 5′-AGACTTTGCTTTCCTTGGTCAGG-3′ and *HPRT* reverse 5′-GTCTGGCTTATATCCAACACTTCG-3′. Primers for *BMI1* (product number QT00052654), *CHGA* (product number QT00025018), *C-MYC* (product number QT00035406), *Cyclin D1* (product number QT00495285), *E-Cadh* (product number QT00080143); *ENO2* (product number QT00084889), *KLF4* (product number QT00061033), *L1CAM* (product number QT00094682), *N-Cadh* (product number QT00063196), *N-Cadh 2* (product number QT00018963), *NeuroD1* (product number QT00203189), *NOTCH1* (product number QT01005109), *NrCAM* (product number QT00006020), *SHH* (product number QT01156799), *SOX9* (product number QT00001498), *SYP* (product number QT00013062) and *TAZ* (product number QT01017996), were purchased from Qiagen. The real-time RT-PCR was done using the MiniOpticon System (Bio-Rad, Hercules, CA, USA) with SYBR Green fluorophore under the following conditions: denaturation at 95°C for 5 min. followed by 40 amplification cycles (denaturation at 95°C for 10 seconds and annealing/extension at 60°C for 30 seconds). Melting curve analysis was done to assess the specificity of PCR products (samples were heated from 65°C to 95°C at a ramp rate of 0.3°C every 5 seconds). The efficiency of reaction for each target was evaluated by amplifying serial dilutions of cDNA. Relative quantification of mRNA was done according to the comparative threshold cycle method with HPRT as calibrator, using the Bio-Rad CFX Manager software. The samples were processed in triplicate, and wells without added cDNA served as negative controls.

Western blotting

Approximately 2.0 × 10^6 cells were collected by low-speed centrifugation (1200 rpm), at room temperature, for 5 minutes. Next, pellets were washed with PBS, at room temperature, and cells were collected by low-speed centrifugation. Then, 1.0 mL of ice cold RIPA Lysis buffer (Thermo Scientific, Waltham, MA, USA) was added, with freshly added Protease and Phosphatase Inhibitors Cocktails (Thermo Scientific), and the cells were incubated on ice for 30 minutes. Then, cells were disrupted and homogenized, transferred to microcentrifuge tubes and centrifuged at 13000 rpm for 10 minutes at 4°C. The supernatants represented the total cell lysates and were transferred to new microfuge tubes.

The following primary anti-human antibodies were used: anti-Nm23-H1 (clone EPR3036) and anti-SOX2 (clone 57CT23.3.4) (both from Abcam); anti-NANOG (clone D73G4, Cell Signaling, Danvers, MA, USA); anti-CDH2/N-Cadh (clone 6G11) and

anti-Neuron-Specific Enolase/ENO2 (clone BBS/NC/VI-H14) (both from Dako); anti-SNAI2/Slug (clone 1A6; OriGene Technologies); anti-Chromogranin A/CHGA (clone E-5), anti-CD44v6 (clone VFF-7), anti-CDH1/E-Cadh, anti-Cyclin D1/Blc-1 (clone DCS-6), anti-Lamin B, anti-NOTCH1, anti-OCT-3/4 (clone C-10) and anti-YAP1 (clone 63.7) (all from Santa Cruz Biotechnology, CA, USA); anti-β-actin (clone AC-15), anti-CDH12/N-Cadh 2, anti-KISS1 (clone 1F7), anti-NrCAM and anti-TAZ (all from Sigma-Aldrich).

Membranes containing proteins were blocked with milk 5X (Sigma-Aldrich) in TBST for 1 hour and, subsequently, probed with primary antibodies and incubated overnight. The membranes were then washed for 3 x 15 minutes with TBST and incubated in diluted (1:5000) horseradish peroxidase conjugated secondary antibodies. The following secondary antibodies were used: rabbit anti-mouse IgG (whole molecule)-Peroxidase and rabbit anti-goat IgG (whole molecule)-Peroxidase (both from Sigma-Aldrich); goat anti-rabbit IgG (H + L)-HRP Conjugate (Bio-Rad). Proteins transferred membranes were washed with TBST for 3 x 15 min and developed with Pierce ECL Western Blotting Substrate (Thermo Scientific).

Supplemental Table S1: SNAI2 gene expression levels in prostate cancer and clinic-pathological parameters[*]

SNAI2 gene espression levels (raw data)[†]	Gleason grade	Age	Stage	PSA[‡] levels (ng/ml)
**Low-grade PCa**				
0,12696	7	73	pT2	< 10
0,62827	5	66	pT2	< 10
0,41028	6	73	pT3	< 10
0,05168	5	69	pT2	< 10
0,33465	5	69	pT2	< 10
0,45012	5	71	pT2	< 10
0,18774	5	73	pT2	< 10
0,00755	5	69	pT3	< 10
0,52350	6	72	pT2	< 10
0,37167	6	58	pT2	< 10
0,55043	6	73	pT2	< 10
0,14776	6	71	pT2	< 10
0,19523	6	72	pT2	< 10
0,21216	5	73	pT2	< 10
0,05512	5	71	pT2	< 10
0,37999	6	60	pT2	< 10
0,33647	6	72	pT2	< 10
0,13521	6	72	pT2	< 10
0,30015	5	72	pT2	< 10
0,50590	5	66	pT2	< 10
0,25341	5	62	pT2	< 10
0,38968	5	70	pT2	< 10

(*Continued*)

SNAI2 gene espression levels (raw data)[†]	Gleason grade	Age	Stage	PSA[‡] levels (ng/ml)
0,49944	8	60	pT2	10–20
0,83174	5	73	pT2	< 10
0,90440	6	72	pT2	< 10
0,56540	5	73	pT2	< 10
0,26201	5	71	pT2	< 10
0,27932	8	69	pT2	10–20
0,85424	7	68	pT2	< 10
0,12561	6	72	pT2	< 10
0,32258	7	71	pT2	< 10
0,90867	6	70	pT2	< 10
0,32916	5	67	pT2	10–20
0,35945	5	71	pT2	< 10
0,64831	5	58	pT2	< 10
**High-grade PCa**				
0,31177	9	73	pT3	> 20
0,04295	8	71	pT2	< 10
0,42904	8	66	pT3	> 20
0,22955	7	72	pT2	< 10
0,60154	8	70	pT3	> 20
0,40018	8	65	pT3	10–20
0,43845	6	65	pT3	< 10
0,51341	8	70	pT2	< 10
0,17468	7	64	pT3	< 10
0,46018	7	71	pT3	< 10
0,62646	8	66	pT3	< 10
0,22264	9	71	pT3	> 20
0,33626	7	63	pT3	< 10
0,05639	9	70	pT3	10–20
0,48000	7	65	pT3	10–20
0,22899	9	68	pT2	10–20
0,17865	7	68	pT3	< 10
0,23131	7	68	pT3	< 10
0,05363	7	73	pT2	10–20
0,37099	7	72	pT2	10–20
0,27122	8	70	pT3	< 10

(*Continued*)

SNAI2 gene espression levels (raw data)[†]	Gleason grade	Age	Stage	PSA[‡] levels (ng/ml)
0,27455	9	71	pT2	10–20
0,12914	7	73	pT2	< 10
0,00064	8	65	pT3	10–20
0,3886	8	73	pT2	> 20
0,38042	9	73	pT3	< 10
0,36265	8	69	pT2	< 10
0,23812	7	65	pT2	< 10
0,26846	8	68	pT2	< 10
0,01796	8	70	pT3	10–20
0,56514	7	68	pT3	10–20
0,10724	7	73	pT2	< 10
0,09634	8	61	pT3	10–20
0,45885	7	71	pT3	< 10
0,0489	9	71	pT2	10–20
0,10034	8	73	pT3	< 10
0,47277	7	71	pT3	10–20
0,37633	7	73	pT2	< 10
0,58272	9	73	pT3	10–20
0,01279	7	59	pT2	< 10
0,36432	7	72	pT3	10–20
0,37644	8	66	pT3	> 20
0,27099	8	70	pT3	10–20
0,43273	7	72	pT3	< 10
0,20660	7	73	pT2	< 10
0,09475	9	68	pT2	> 20
0,44685	7	63	pT3	< 10
0,36024	8	70	pT3	> 20
0,46159	8	61	pT3	< 10
0,52655	7	58	pT2	10–20
0,32509	8	72	pT3	> 20
0,19388	7	70	pT2	< 10
0,30523	9	67	pT3	> 20
0,10516	7	73	pT3	10–20
0,09186	7	73	pT3	< 10
0,18820	8	64	pT3	< 10

(*Continued*)

SNAI2 gene espression levels (raw data)†	Gleason grade	Age	Stage	PSA‡ levels (ng/ml)
0,18652	6	73	pT2	< 10
0,50866	9	64	pT3	< 10
0,01382	8	70	pT2	< 10
0,39367	8	68	pT2	< 10
0,10338	7	72	pT2	< 10
0,08744	8	71	pT3	< 10
0,13443	8	67	pT2	> 20
0,15732	7	71	pT3	10–20
0,42991	8	69	pT3	10–20
0,35925	7	73	pT3	10–20
0,36398	7	69	pT3	< 10

*Each line report the SNAI2 gene expression level of a single patient with the corresponding clinical and pathological parameters. The patients are divided into two categories, low- vs high-grade PCa, on the basis of the Gleason grade of cancer foci used for microdissection.
†Gene expression levels are relative to XpressRef™ Universal Total RNA from SABiosciences.
‡Prostate-Specific Antigen.

Supplemental Table S2: Mann-Whitney *U* test*

Clinical parameters	Ua	Z†	*p* (Two-tailed)
**Gleason Grade**			
G5	832	–0,66	0,5093
G6	721	–1,51	0,131
G7	1036	0,6	0,5485
G8	958	0,41	0,6818
G9	433	0,72	0,4715
**Age**			
58–63	612	–1,2	0,2301
64–68	1087	–1,19	0,234
69–73	1435	–1,85	0,0643
**Stage**			
pT2 vs pT3	1357	–0,54	0,5892
**PSA levels**			
< 10	1133	0,27	0,7872
10–20	876	0,47	0,6384
>20	521	–0,22	0,8259

*The Mann–Whitney U probability test was applied with $p < 0.05$ as the significance cut-off.
†Standard score.

Supplemental Table S3: χ^2 test*

**Clinical parameters**	**Chi**	**df†**	***p***
Gleason grade	3,8	4	0,4337
Age	3,17	2	0,2049
Stage	1	1	0,3173<br>(Yates Chi-square corrected for continuity)
PSA levels	0,11	2	0,9465

*The χ^2 test was applied with $p < 0.05$ as the significance cut-off. We assessed the probability that clinic-pathological parameters could be associated with SNAI2 expression levels over or under the median value.
†Degrees of freedom.
